# Impact of systemic disease on CNS disease control after stereotactic radiosurgery to breast cancer brain metastases (The SYBRA Study)

**DOI:** 10.1038/s41523-024-00673-z

**Published:** 2024-08-02

**Authors:** Alex Schick, Sara Hardy, Myla Strawderman, Dandan Zheng, Michael Cummings, Michael T. Milano, Allison Magnuson, Jacqueline Behr, Sarah Sammons, Kenneth Usuki, Nimish Mohile, Ruth O’Regan, Carey K. Anders, David Hicks, Ajay Dhakal

**Affiliations:** 1 Division of Hematology/Oncology, Department of Medicine & Wilmot Cancer Institute, University of Rochester Medical Center, Rochester, NY USA; 2 Department of Radiation Oncology, Wilmot Cancer Institute, University of Rochester Medical Center, Rochester, NY USA; 3 Department of Biostatistics and Computational Biology, University of Rochester Medical Center, Rochester, NY USA; 4 Division of Neuro-Oncology, Department of Neurology & Wilmot Cancer Institute, University of Rochester Medical Center, Rochester, NY USA; 5 Department of Medical Oncology, Dana-Farber Cancer Institute, Harvard Medical School, Boston, MA USA; 6 Division of Medical Oncology, Duke Cancer Institute, Duke University Medical Center, Durham, NC USA; 7 Department of Pathology and Laboratory Medicine, University of Rochester Medical Center, Rochester, NY USA

**Keywords:** Breast cancer, Metastasis

## Abstract

The objective of the study is to assess impact of systemic disease (SD) status on overall survival and brain metastasis (BM) control, adopting a novel landmark approach to categorize SD among breast cancer (BC) patients. This single institution retrospective study included BCBM patients who have received stereotactic radiosurgery (SRS) to brain. Separate endpoints [CNS failure-free survival (cFFS), overall survival (OS)] were analyzed from each Landmark (LM): LM1 (3-months), LM2 (6-months). Patients were categorized into early and non-early progression (EP, NEP) groups depending on SD status before LMs. Median survivals from LM were assessed with Kaplan Meier plots, compared with Log-Rank test. EP was associated with worse median cFFS and OS vs NEP in both LM analyses (cFFS- LM1: 3.6 vs. 9.7 months, *p* = 0.0016; LM2: 2.3 vs. 12.5 months, *p* < 0.0001; OS- LM1: 3.6 vs. 24.3 months, *p* < 0.0001; LM2: 5.3 vs. 30.2 months, *p* < 0.0001). In multivariate analyses, EP was associated with shorter cFFS [LM1: Hazard Ratio (HR) with 95% confidence interval (CI) 3.16, 1.46–6.83, *p* = 0.0034; LM2: 5.32, 2.33–12.15, *p* = <0.0001] and shorter OS (LM1: HR with 95% CI 4.28, 1.98–9.12, *p* = 0.0002; LM2: 7.40, 3.10–17.63, *p* = <0.0001) vs NEP. Early systemic disease progressions after 1st SRS to brain is associated with worse cFFS and OS in patients with BCBM.

## Introduction

Many patients with metastatic breast cancer (MBC) develop brain metastasis, which is associated with a poor prognosis^1,2^. Though the overall survival of patients with breast cancer brain metastases has improved over the last three decades, it remains grim^3^. Metastasis to extra-cranial sites are mainly treated with systemic therapy. However, brain metastases are mainly treated with local therapy (radiation or, less commonly, surgery) due to the historical lack of efficacy of traditional systemic therapies in the Central Nervous System (CNS). Currently, stereotactic radiosurgery (SRS) is the treatment of choice for up to 4 brain metastases and is a reasonable option for patients with more than four brain metastases^4^.

Various studies have investigated the factors associated with the failure of CNS disease control after SRS to brain metastases^5–7^. These studies have inconsistently identified systemic disease status as a significant predictor of CNS disease control, and have two major limitations. First, these studies include patients with various primary tumor types. Different tumor types are associated with different biology, systemic treatments, and survival. Second, these studies have assessed the systemic disease status at the time of SRS. As the CNS failure events occur *after* SRS in these studies, it is essential to have a more dynamic assessment of systemic disease status *after and not at the time of* the SRS administration. Moreover, systemic therapies are often changed to those with better CNS activity at the time of development of brain metastasis. This could lead to a change in systemic disease status: a systemic disease considered “progressive” at the study entry could subsequently respond to a new systemic therapy initiated after SRS. In the same way, a “stable” systemic disease at the time of study entry may progress after SRS due to a change in the systemic therapy geared more towards CNS disease control.

Similarly, prior studies have identified systemic disease status as prognostic for survival among patients with brain metastasis^3,8,9^. Systemic disease status has been included in the updated standard prognostic index [The Breast Graded Prognostic Assessment (GPA)], commonly used to assess the prognosis of patients with brain metastases from breast cancer^3^. However, the systemic disease status is categorized into “absent” vs. “present” at the time of brain metastasis diagnosis in the Breast GPA model. This categorization is too simplistic. First, the majority of patients with breast cancer brain metastases will have systemic disease present. Second, simply classifying systemic disease as “present” fails to differentiate a rapidly progressive systemic disease from a systemic disease that has been stable for years. Here, we report a novel study (The SYBRA study) to more accurately assess the impact of systemic disease status on CNS disease control and overall survival of patients with brain metastases from breast cancer after local treatment.

## Results

Of 163 patients whose medical records were screened, 104 were eligible and registered in the study. Data were locked on August 8, 2023, after which no updates were made to the survival or CNS events. As this is a retrospective study, observation time is described using “reverse censoring” (alive patients are complete observation times, whereas patients who died are censored at death). Median observation time was 57 months, with a maximum of 152 months following 1^st^ SRS completion. Table 1 shows patients, disease, and treatment characteristics of the entire eligible cohort, CNS failure free survival evaluable patients at LM1 and LM2, and comparisons between the Early Progression vs. Non-Early Progression groups in each LM analysis. No statistically significant differences between Early Progression and Non-Early Progression groups by factors in Table 1 at either LM were observed.

**Table 1:** Description of overall sample and by systemic failure status at each landmark

Patient characteristics at 1^st^ SRS	Eligible sample *N* = 104	CNS failure free survival analysis from landmark 1			CNS failure free survival analysis from landmark 2		
		Total evaluable *N* = 81	Non-early progression *N* = 67	Early progression *N* = 14	Total evaluable *N* = 65	Non-early progression *N* = 49	Early progression *N* = 16
Age, years							
Mean (SE)	58 (1)	59 (1)	58 (1)	65 (3)	60 (1)	60 (1)	58 (4)
< 60	51 (49)	37 (46)	33 (49)	4 (29)	28 (43)	20 (40)	8 (50)
≥ 60	53 (51)	44 (54)	34 (51)	10 (71)	37 (57)	29 (59)	8 (50)
*P*-value^a^			P = 0.16			*P* = 0.52	
Race							
Asian	5 (5)	4 (5)	4 (5)	0	4 (6)	3 (6)	1 (6)
Black	11 (11)	10 (12)	10 (15)	0	8 (12)	7 (14)	1 (6)
White	87 (84)	67 (83)	53 (79)	14 (100)	53 (82)	39 (80)	14 (88)
Unknown	1 (1)	0	0	0	0	0	0
P-value^a^			P = 0.17			*P* = 0.85	
Hispanic							
Yes	1 (1)	1 (1)	1 (1)	0	0	0	0
No	99 (95)	77 (95)	63 (94)	14 (100)	62 (95)	47 (96)	15 (94)
Unknown	4 (4)	3 (4)	3 (4)	0	3 (5)	2 (4)	1 (6)
KPS							
90–100	42 (40)	37 (46)	30 (45)	7 (50)	33 (51)	25 (51)	8 (50)
70–85	48 (46)	37 (46)	30 (45)	7 (50)	26 (40)	18 (37)	8 (50)
≤ 60	12 (12)	5 (6)	5 (7)	0	4 (6)	4 (8)	0
Unknown	2 (2)	2 (2)	2 (3)	0	2 (3)	2 (4)	0
P-value^a^			P = 0.75			*P* = 0.50	
Hormone Receptors							
ER + / HER2-	45 (43)	35 (43)	27 (40)	8 (57)	25 (38)	17 (35)	8 (50)
ER- / HER2-	23 (22)	16 (20)	12 (18)	4 (29)	11 (17)	8 (16)	3 (19)
HER2+	33 (32)	27 (33)	25 (37)	2 (14)	27 (42)	23 (47)	4 (25)
Unknown	3 (3)	3 (4)	3 (4)	0	2 (3)	1 (2)	1 (6)
P-value^a^			*P* = 0.25			*P* = 0.38	
Cancer Subtype							
Lumina A	45 (43)	35 (43)	27 (40)	8 (57)	25 (38)	18 (36)	8 (50)
Lumina B	16 (15)	13 (16)	12 (18)	1 (7)	13 (20)	11 (22)	2 (13)
HER2 Type	17 (16)	14 (17)	13 (19)	1 (7)	14 (22)	12 (24)	2 (13)
Basal	23 (22)	16 (20)	12 (18)	4 (29)	11 (17)	8 (16)	3 (19)
Unknown	3 (3)	3 (4)	3 (4)	0	2 (3)	1 (2)	1 (6)
P-value^a^			P = 0.41			P = 0.57	
Brain Mets at SRS							
1	42 (40)	33 (41)	27 (40)	6 (43)	30 (46)	23 (47)	7 (44)
2–3	31 (30)	23 (28)	19 (28)	4 (29)	18 (28)	12 (24)	6 (38)
> = 4	30 (30)	24 (30)	20 (30)	4 (29)	16 (26)	14 (29)	2 (13)
Unknown	1 (1)	1 (1)	1 (1)	0	1 (2)	0	1 (6)
P-value^a^			P > 0.99			P = 0.17	
Fraction Number							
1	44 (42)	39 (48)	29 (43)	10 (71)	31 (48)	20 (41)	11 (69)
3	41 (40)	26 (32)	23 (35)	3 (21)	18 (28)	16 (33)	2 (13)
5	16 (15)	13 (16)	12 (18)	1 (7)	13 (20)	11 (22)	2 (13)
missing	3 (3)	3 (4)	3 (4)	0	3 (5)	2 (4)	1 (6)
P-value^a^			P = 0.27			P = 0.21	
Planning Target Volume							
mean (SE)	12.4 (1.9)	9.1 (1.2)	9.3 (1.3)	8.5 (3.4)	9.5 (1.4)	9.9 (1.6)	8.2 (3.3)
median	5.3	4.7	5.0	2.3	5.0	6.8	3.1
(IQR)	(1.7, 14.0)	(1.6,12.6)	(1.6, 13.2)	(1.2, 6.2)	(1.6, 13.6)	(2.0, 13.9)	(1.2, 6.9)
missing	5	5	5	0	5	3	2
P-value^b^			P = 0.39			P = 0.29	
Systemic Therapy *After* 1^st^ SRS							
None	12 (12)	6 (7)	6 (9)	0	5 (8)	5 (10)	0
Chemo +/- endocrine / immunotherapy	42 (40)	34 (42)	25 (37)	9 (64)	22 (34)	13 (27)	9 (56)
Targeted agents	28 (27)	22 (27)	20 (30)	2 (14)	19 (29)	17 (35)	2 (13)
Her2 directed +/- chemotherapy	16 (15)	14 (17)	12 (18)	2 (14)	14 (22)	11 (22)	3 (19)
Antibody drug conjugate	6 (6)	5 (6)	4 (6)	1 (7)	5 (8)	3 (6)	2 (13)
P-value^a^			P = 0.34			P = 0.10	

^a^Chi-square test compares the proportions between groups defined by systemic disease status at the landmark.

^b^Wilcoxon Rank Sum test compares the median between groups defined by systemic disease status at the landmark.

### LM-1 (3 month post 1^st^ SRS) analyses

**Fig. 1:**
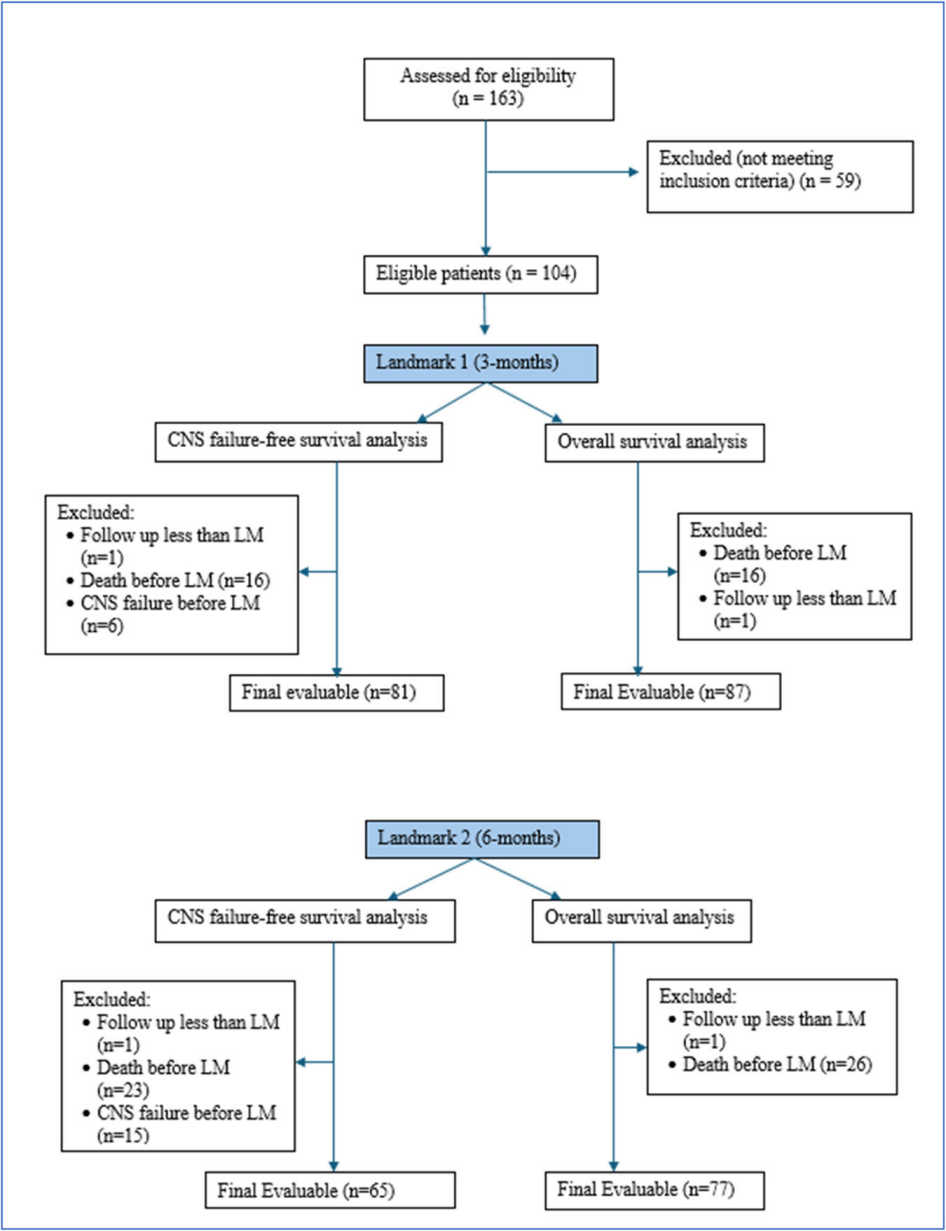
**CONSORT-Like Diagram of the Study**.

Among the 104 eligible patients, 23 were excluded from the primary analysis at the first landmark. (Supp. Table 1). One of the 23 was excluded because that patient had not been observed until the first landmark. The remaining 22 were excluded due to CNS failure (*n* = 6) or death (*n* = 16) before landmark (Fig. 1).

**Fig. 2:**
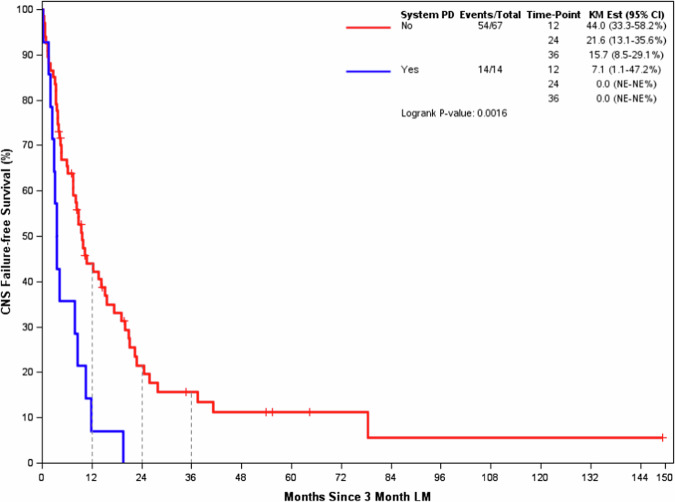
Landmark 1 (3-month post 1^st^ stereotactic radiosurgery) CNS Failure-Free Survival. CNS Failure Free Survival among Early Progression group (Blue line) was significantly shorter than Non-early Progression group (Red line) from 3-month Landmark.

Of the 81 analyzed at LM1, 14 patients developed systemic disease progression before the landmark (Early Progression -LM1). All 14 of the patients in the Early Progression -LM1 group had CNS failure free survival events (6 CNS failure and 8 deaths). Sixty-seven patients had not experienced a systemic failure before the landmark (Non-Early Progression -LM1). Of these, 54 had a CNS failure free survival event by the time of this analysis (33 CNS failure and 21 deaths). Figure 2 shows K-M estimated CNS failure free survival. The median CNS failure free survival [95% Confidence Interval (CI)] from LM1 for Early Progression -LM1 was 3.6 months (2.9–11.9) compared to 9.7 months (7.4–15.5) in the Non-Early Progression -LM1 group (*p* = 0.0016).

In an exploratory analysis, the proportion of CNS failure among all events (CNS failure and deaths) between the two groups was not statistically significant (Supp. Table 2). Among 6 patients with CNS failure in the Early Progression -LM1, there were 0 local CNS relapses (progression of the prior radiated brain metastases), 2 distant CNS relapses (development of new brain metastases), 2 distant plus local CNS relapses, and 2 cases with the type of CNS relapse unknown. Among 33 patients with CNS failure in the Non-Early Progression -LM1 Group, 10 had local CNS relapse; 9 had distant CNS relapses, 4 distant plus local CNS relapse, and 10 cases of unknown CNS relapse.

Table 2 shows Cox proportional hazards model of systemic disease progression before LM1 vs. not with CNS failure free survival from LM1, adjusting for additional covariates. Table 2 uses 70 of the 81 patients shown in Fig. 2 with complete covariate information. We observed 58 events, 33 CNS failures, and 25 deaths in this subset. Early system disease progression before LM1 was statistically associated with the time to CNS failure or death after the LM [Hazard Ratio (HR) with 95% CI 3.16, 1.46–6.83; *p* = 0.0034] after adjusting for all other factors included in the model.

**Table 2:** Adjusted association with time to CNS failure or death from Landmarks post 1^st^ stereotactic radiosurgery

Patient Characteristics	Hazard Ratio	95% CI	*P*-value
**Landmark 1**			
Systemic PD prior to LM1 vs not.	3.16	1.46, 6.83	0.0034
Age (per decade)	0.80	0.63, 1.01	0.0584
Race (white vs not white)	1.00	0.43, 2.32	0.9898
KPS 90–100 vs <90	0.49	0.27, 0.88	0.0186
ER + /Her2- vs Her2+	1.80	0.91, 3.53	0.0904
ER-/Her2- vs Her2+	2.77	1.23, 6.25	0.0142
Brain Mets at SRS1 ( > 1 vs 1)	2.40	1.30, 4.45	0.0052
PTV (per log)	0.98	0.80, 1.19	0.8173
Fractions (1 vs 3 or 5)	0.82	0.45, 1.50	0.5285
**Landmark 2**			
Systemic PD prior to LM2 vs not.	5.32	2.33, 12.15	<0.0001
Age (per decade)	0.84	0.65, 1.10	0.2070
Race (white vs not white)	0.81	0.30, 2.20	0.6786
KPS 90–100 vs <90	0.85	0.43, 1.67	0.6290
ER + /Her2- vs Her2+	0.82	0.37, 1.81	0.6236
ER-/Her2- vs Her2+	1.26	0.48, 3.28	0.6336
Brain Mets at SRS1 ( > 1 vs 1)	2.18	1.05, 4.52	0.0367
PTV (per log)	1.05	0.83, 1.33	0.6932
Fractions (1 vs 3 or 5)	0.72	0.35, 1.45	0.3514

**Fig. 3:**
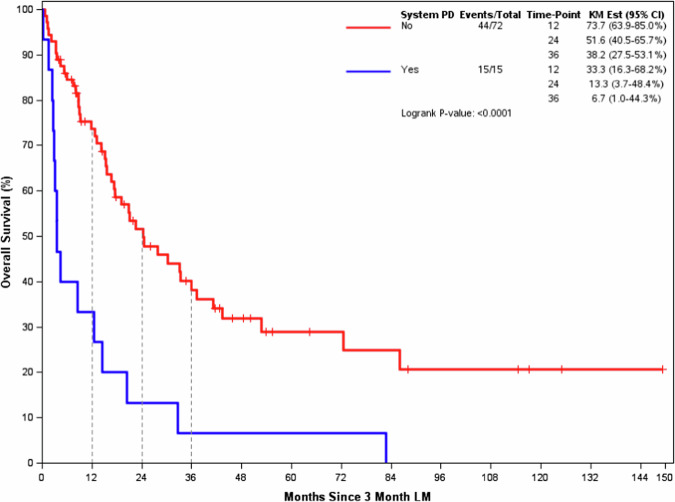
Landmark 1 (3-month post 1^st^ stereotactic radiosurgery) Overall Survival. Overall Survival among Early Progression group (Blue line) was significantly shorter than Non-early Progression group (Red line) from 3-month Landmark.

Six additional patients are included in the OS analysis from LM1 (See Supp. Table 1). The median OS (95% CI) from LM1 was 3.6 months (2.9–20.5) in the Early Progression -LM1 Group compared to 24.3 months (17.2–41.2) in the Non-Early Progression -LM1 Group (*p* < 0.001). See Fig. 3 for the K-M plot. Table 3 shows proportional hazards regression using 76 of 87 patients shown in Fig. 3, with complete covariate information. In this model, 49 deaths were observed. System disease progression before the LM1 was statistically associated with the time to death (HR with 95% CI 4.28, 1.98–9.12; *p* = 0.0002) after adjusting for all other factors included in the model.

**Table 3:** Adjusted association with time to death from Landmarks post 1^st^ stereotactic radiosurgery

Patient Characteristics	Hazard Ratio	95% CI	*P*-value
**Landmark 1**			
Systemic PD prior to LM1 vs not.	4.28	1.98, 9.12	0.0002
Age (per decade)	0.82	0.63, 1.06	0.1264
Race (white vs not white)	0.96	0.38, 2.43	0.9257
KPS 90–100 vs <90	0.52	0.26, 1.01	0.0537
ER + /Her2- vs Her2+	2.08	1.01, 4.25	0.0456
ER-/Her2- vs Her2+	2.28	0.92, 5.60	0.0736
Brain Mets at SRS1 ( > 1 vs 1)	1.92	1.01, 3.62	0.0455
PTV (per log)	1.02	0.83, 1.26	0.8504
Fractions (1 vs 3 or 5)	1.14	0.55, 2.36	0.7206
**Landmark 2**			
Systemic PD prior to LM2 vs not.	7.40	3.10, 17.63	<0.0001
Age (per decade)	0.87	0.66, 1.14	0.3004
Race (white vs not white)	0.56	0.21, 1.50	0.2451
KPS 90–100 vs <90	0.55	0.26, 1.18	0.1267
ER + /Her2- vs Her2+	1.23	0.57, 2.66	0.5966
ER-/Her2- vs Her2+	1.06	0.38, 2.95	0.9095
Brain Mets at SRS1 ( > 1 vs 1)	1.59	0.78, 3.25	0.2066
PTV (per log)	1.15	0.90, 1.46	0.2625
Fractions (1 vs 3 or 5)	1.22	0.54, 2.78	0.6366

### LM-2 (6 month post 1^st^ SRS) analyses

Among the 104 eligible patients, 39 were excluded from the primary analysis at LM2, leaving 65 patients for the CNS failure free survival analysis. One of the 39 patients was excluded because that patient had not been observed until the second landmark (55 days of follow-up as of the data lock). The remaining 38 were excluded due to CNS failure (*n* = 15) or death (*n* = 23) before the landmark (Supplementary Table 1).

**Fig. 4:**
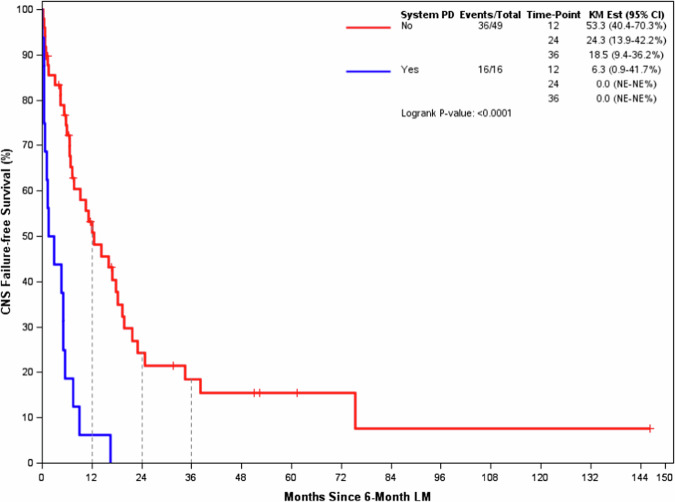
Landmark 2 (6-month post 1^st^ stereotactic radiosurgery) CNS Failure-Free Survival. CNS Failure Free Survival among Early Progression group (Blue line) was significantly shorter than Non-early Progression group (Red line) from 6-month Landmark.

All 16 patients in the Early Progression -LM2 group had an event (7 CNS failures, 9 deaths). Of 49 patients in the Non-Early Progression -LM2 group, 36 had an event by the time of this analysis (23 CNS failures, 13 deaths). Figure 4 shows CNS failure free survival among the groups with K-M plots. Median CNS failure free survival from LM2 was 2.3 months (95% CI 0.6–7.5) in the Early Progression -LM2 group compared to 12.5 months (95% CI 7.7–19.7) in the Non-Early Progression -LM2 group (*p* < 0.0001).

In an exploratory study, the proportion of CNS failure among all events (CNS failure and deaths) between groups was not statistically significant (Supp. Table 2). Among 7 patients who had CNS failure in the Early Progression -LM2 group, there were 0 local CNS relapses, 2 distant CNS relapses, 1 distant plus local CNS relapse, and 4 cases with the type of CNS relapse unknown. Among 23 patients with CNS failure in the Non-Early Progression -LM2 group, 10 had local CNS relapse, 5 had distant CNS relapse, 3 distant plus local CNS relapses, and 5 cases with the type of CNS relapses unknown.

Table 2 shows the results of a proportional hazards regression using 55 patients of the 65 patients shown in Fig. 4 with complete covariate information for all potential predictors of interest. This model is based on 43 events: 24 were CNS failures, and 19 were deaths. System disease progression before LM2 is statistically associated with a shorter time to CNS failure or death (HR with 95% CI 5.32, 2.33–12.15; *p* < 0.0001) after adjusting for all other factors included in the model.

**Fig. 5:**
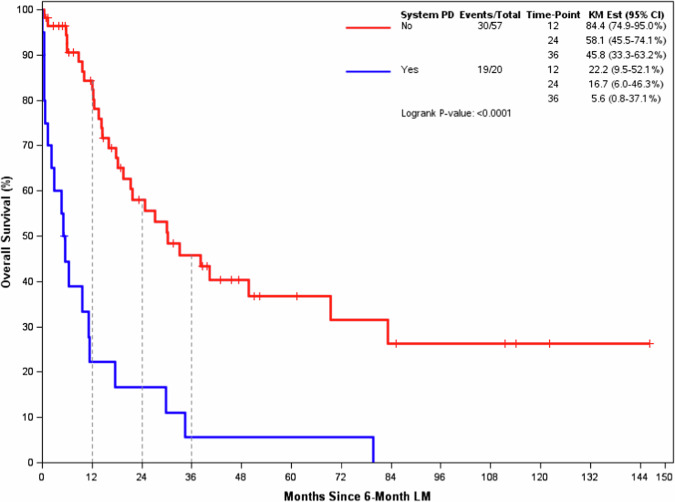
Landmark 2 (6-month post 1^st^ stereotactic radiosurgery) Overall Survival. Overall Survival among Early Progression group (Blue line) was significantly shorter than Non-early Progression group (Red line) from 6-month Landmark.

Of the 20 patients in the Early Progression -LM2 group, 19 (95%) had died. Of the 57 patients in the Non-Early Progression -LM2 group, 30 (60.7%) had died. Figure 5 shows overall survival between two groups with K-M plots. The median overall survival from LM2 was 5.3 months (2.2–17.5) in the Early Progression -LM2 Group compared to 30.2 months (95% CI 21.3–83.1) in the Non-Early Progression -LM2 Group (*p* < 0.0001).

Table 3 shows the results of a proportional hazards regression using 67 patients of the 77 patients shown in Fig. 5 with complete covariate information for all potential predictors of interest. Among the 67 patients, 40 deaths were observed. System disease progression before the LM at six months is statistically associated with the time to death (HR with 95% CI 7.40, 3.10–17.63; *p* < 0.0001) after adjusting for all other factors included in the model.

### Exploratory analyses based on breast cancer subtypes

Supplementary Figs. 2–5 compare the CNS failure free survival and overall survival between the Early Progression group vs. Non-Early Progression group subdivided into three breast cancer subtypes: estrogen receptor-positive (ER + ), HER2 non amplified (HER2-) group, HER2 + group, and the triple negative (ER-/HER2-) group. In the ER + /HER2- breast cancer with brain metastasis, median CNS failure free survival and overall survival from both LMs were shorter among the Early Progression group compared to the Non-Early Progression group. In the HER2+ subtype, the CNS failure free survival from LM2 and overall survival from both LM1 and LM2 were shorter among the Early Progression group than the Non-Early Progression group. The CNS failure free survival from LM1 in this subtype were not significantly different between the groups. In the triple negative subtype, CNS failure free survival and overall survival were not significantly different between groups from both landmarks.

### Exploratory cause of death analysis

Supplementary Tables 3 compare the causes of death among the Early Progression vs. Non-Early Progression groups in LM1 and LM2 analysis, respectively.

### Exploratory analysis of radiological CNS progression

As the study defined CNS failure as the evidence of subsequent local CNS therapy, exploratory analysis (Supplementary Table 4) was done to investigate the percentage of those patients who had radiological evidence of CNS progression but were not counted as CNS Failure because they were not treated with subsequent local CNS therapy for various reasons. The proportion of patients (among those who didn’t have CNS failure per study definition) who had radiological evidence of CNS progression was not different between the Early Progression and Non-Early Progression groups. In an additional exploratory analysis, where radiological evidence of CNS progression was also considered a CNS failure free survival event (in addition to the study defined events- subsequent CNS radiation or death), the CNS failure-free survival remained significantly shorter in the Early Progression group vs. Non-Early Progression group from both LMs (Supp Fig. 6). Further, cause-specific analyses were performed (Supp Fig. 7). Here, the event was defined as subsequent CNS radiation or radiological evidence of CNS progression. Subjects who died prior to having events were censored at the time of death. There was no significant difference in time to CNS progression among Early and Non-Early Progression groups from LM1. However, the Early Progression group was associated with a significantly shorter time to CNS progression than the Non-Early Progression group from LM2.

## Discussion

We observed significantly worse CNS failure-free survival and overall survival among patients with early systemic disease progression (within the subsequent 3 or 6 months post-SRS) compared to patients who did not have progression within these LMs. These data show the critical need for systemic disease control among patients who have received SRS to brain metastases. Among those with early systemic disease progression, more than half (57% in LM1 analysis, 56% in LM2) of CNS failure free survival events were dead before CNS progression, suggesting systemic disease is a dominant driver of survival for many patients. Only 14% and 6% of the CNS failure free survival events had a local relapse (LM1: 0% local only + 14% local plus distant; LM2: 0% local + 6% local plus distant) among those with early systemic disease progression. These percentages are numerically higher among those without early systemic disease progression [LM1: 25% (18% + 7%); LM2: 35% (27% + 8%)]. These data suggest SRS provides reasonable disease control on the treated brain metastases among those with early systemic disease progression compared to those without. However, it is likely that significantly shorter overall survival in the early systemic disease progression group does not allow enough time for the patients to have CNS relapse. These data may suggest greater need to prioritize systemic disease control.

Nevertheless, the number of different CNS failure events is small, and these data are hypothesis-generating rather than confirmatory. Interestingly, more than one-third (40% LM1; 38% LM2) of the CNS failure free survival events among the patients without early systemic disease progression were death without CNS failure. This suggests that a significant percentage of patients who did not have early systemic disease progression after their 1^st^ SRS to brain metastases still were dying from systemic disease without any subsequent CNS failure. These data may suggest the need for continuous systemic disease control for brain metastasis patients who initially experienced systemic disease control.

Guidelines and expert opinions favor systemic therapy with better CNS activity while treating breast cancer with brain metastasis^10–13^. Sometimes, systemic therapy with the best CNS efficacy may not be the systemic therapy with the best systemic disease activity. For instance, taxanes and anthracyclines, considered to be the most effective chemotherapeutic agents against breast cancer, are not on the list of the recommended systemic therapy regimens for breast cancer brain metastases in the National Comprehensive Cancer Network (NCCN) guidelines because of their weak CNS activity data^10,14^. Our study demonstrates the significant impact of systemic disease control on overall and CNS-specific survival and questions the current trend of choosing systemic therapy which is more focused on CNS control as opposed to being primarily focused on systemic disease control. Similarly, as more systemic therapies with CNS efficacy have developed, current American Society of Clinical Oncology (ASCO) guidelines provide the option of delaying local radiation and choosing systemic therapy with CNS efficacy in asymptomatic brain metastases based on panel consensus^4^. This approach may provide control of both CNS and systemic disease with a single cancer therapy and avoid side effects of CNS radiation. However, it is unclear if a deferred CNS radiation plus a systemic therapy with the best intracranial activity is superior to an approach of upfront CNS control with SRS plus the best systemic disease activity (without worrying about CNS activity) in terms of CNS-specific and overall survival.

An ongoing phase II multicenter study investigates the efficacy of genomically guided systemic therapy for brain metastasis^15^. The systemic therapy in this study is based on genomic alterations in the brain metastasis tissue. Studies have suggested different genomic alterations in brain metastasis compared to extra-CNS metastasis^16^. Separate CNS and extra-CNS tumor response assessments will be done in this study. Even though the primary endpoint of this study is the antitumor activity of targeted therapies in the CNS, it will be equally interesting to understand how systemic disease control or lack thereof may influence overall survival.

The SYBRA study has limitations. This study is a single institution study. The results are hypothesis-generating and warrant validation on a larger multicenter data set, ideally in a prospective manner. Secondly, subsequent local CNS therapy (surgery or radiation) was used as a surrogate for CNS failure. This retrospective study did not use standard tumor response assessments, such as RECIST 1.1 or BM-RANO. As such, subsequent local CNS therapy was considered a more objective and accurately obtainable data point compared to the date of radiological progression. Nevertheless, not using standard tumor response assessments may impact the study’s reliability. CNS failure events for patients with true radiological progression may be underestimated in our analysis. Predicting early systemic disease progression among patients with breast cancer brain metastasis was not the objective of the SYBRA study. However, the result of this study warrants future studies to predict early systemic disease progression upfront and investigate role of systemic therapy escalation for these select patients to achieve better systemic disease control. An additional limitation of the study is that due to the limited number of events observed in the sample, we were not able to explore potential interactions between the factors included in the model. Those analyses will need to be explored in a larger data set.

Multiple targeted systemic cancer therapies have been developed in the last decade with potential intracranial activity. At the same time, the standard CNS radiation treatment has largely moved away from whole-brain radiation therapy to SRS. The SYBRA study has suggested that systemic disease is the major driver of overall survival among patients with breast cancer brain metastasis after SRS. Ultimately, the effect of systemic disease control on CNS outcomes and overall survival should be assessed in disease-specific randomized prospective trials. For patients receiving SRS for CNS disease control, future clinical trials should compare an SRS plus a systemic disease choice with the best chance of systemic disease control vs. SRS plus a systemic disease choice with the best CNS activity. In instances where the systemic therapy with the best chance of systemic disease control is the systemic therapy with the most promising CNS activity, the CNS-specific and overall survival with deferred vs. upfront SRS should be compared.

In conclusion, the SYBRA study compared the CNS and overall survival outcomes of patients with breast cancer brain metastasis between those with and without early systemic disease progression after their first SRS to brain metastasis. The study has shown that patients with early systemic disease progression have significantly worse CNS failure-free survival and overall survival compared to those without early systemic disease progression. The small sample size in the study may affect the robustness of the study results. These findings should be validated in a larger, multi-institutional cohort, using standardized tumor response assessments.

## Methods

### Patient population

Patients with brain metastases from MBC who have undergone at least one course of SRS therapy were identified at the Wilmot Cancer Institute’s Department of Radiation Oncology through an existing database and medical record queries. Key inclusion criteria were: 1. Patients with at least one brain metastasis from MBC. 2. Have received first or only SRS for brain metastases between January 1, 2010, and July 1, 2023. Patients with radiologic or cytologic evidence of leptomeningeal disease were excluded from the study. University of Rochester Research Subjects Review Board (RSRB) approved this study (STUDY00008348: The SYBRA Study). The need for obtaining informed consent was waived by RSRB due to retrospective chart review nature of this study.

### Design

We completed a retrospective analysis comparing the CNS failure-free survival, and overall survival of patients with brain metastases from MBC with systemic disease progression classified as early progression vs. non-early progression after their 1^st^ brain SRS, using two separate landmark (LM) analyses.

1. 3-month LM Analysis: Compare CNS failure free survival and overall survival after the 1^st^ SRS to the brain among MBC patients whose systemic disease progresses vs. doesn’t progress before three months (90 days, LM-1) from the last day of treatment of the 1^st^ SRS [Early Progression Group-LM1 vs. Non-Early Progression Group-LM1].
2. 6-month LM Analysis: Compare CNS failure free survival and overall survival after the 1^st^ SRS to the brain among MBC patients whose systemic disease progresses vs. doesn’t progress before six months (180 days, LM-2) from the last day of the 1^st^ SRS [Early Progression Group-LM2 vs. Non-Early Progression Group-LM2].

CNS failure was defined as evidence of any subsequent local treatment to the brain (any radiation treatment to the brain or surgical resection) after the 1^st^ SRS. CNS failure free survival was defined as duration between LM and CNS failure or death, whichever comes first. Overall survival was defined as the duration between LM and death. The primary endpoints are CNS failure free survival from each landmark, while overall survival from each landmark are secondary endpoints. Patients with CNS failure before LM were excluded from the CNS failure free survival analysis but included in the overall survival analysis. Patients who had died before LM were excluded from both analyses. The systemic disease progression was determined per the treating physicians’ assessment based on radiological data. Supplementary Fig. 1 shows the study groups in context of landmarks, SRS and CNS Failure Free Survival events.

### Statistical analysis

To address the issue of immortal time bias introduced by comparing the time to an event of interest (CNS failure or death) from SRS by an intervening event, like systemic disease status after SRS, a landmark analysis approach was adopted. This method describes the time to the event of interest from the landmark conditional on the systemic disease status at the landmark^17–19^. The median CNS failure free survival and overall survival from the landmark among the Early Progression and Non-Early Progression groups were assessed with Kaplan Meier plots and compared with the Log-Rank test. CNS failure free survival and overall survival rates at 1, 2, and 3 years were reported by systemic disease progression status with 95% confidence intervals (CI). Cox proportional regression models adjusted the association between the systemic disease status and outcomes for other important variables. Comparisons between Early Progression and Non-Early Progression groups for categorical patient characteristics were evaluated with Chi-square test or Fisher’s Exact test and by the Wilcoxon Rank Sum test for continuous characteristics.

### Supplementary information

Supplementary material

## Data Availability

De-identified data set will be made available upon reasonable request to the corresponding author.
