# Supplementary material for: Impact of systemic disease on CNS disease control after stereotactic radiosurgery to breast cancer brain metastases (The SYBRA Study)
Source: NPJ Breast Cancer. 2024 Aug 2;10:69. doi: 10.1038/s41523-024-00673-z (PMC11297231; doi:10.1038/s41523-024-00673-z)

Supplementary Table 1: Summary of excluded patients from primary and secondary analysis in landmarks.

Exclude from CNS failure free survival Analysis	CNS failure free survival Exclusion Reason	Exclude from Overall Survival Analysis	Overall Survival Exclusion Reason	Frequency	Cumulative Frequency
**Landmark 1**					
NO		NO		81	81
YES	CNS Failure Before LM	NO		6	87
YES	Died Before LM	YES	Died Before LM	16	103
YES	Follow-Up < LM Unknown system Disease status	YES	Follow-Up < LM Unknown system Disease status	1	104
**Landmark 2**					
NO		NO		65	65
YES	CNS Failure Before LM	NO		12	77
YES	CNS Failure Before LM	YES	Died Before LM	3	80
YES	Died Before LM	YES	Died Before LM	23	103
YES	Follow-Up < LM Unknown system Disease status	YES	Follow-Up < LM Unknown system Disease status	1	104

Supplementary Table 2: Type of CNS failure free survival events in landmarks.

Event	Early Systemic Disease Progression Group	Non-Early Systemic Disease Progression Group	Chi Square Test[1]
**Landmark 1**			
**Number (N)**	**14**	**54**	
Any CNS Progression	6 (43)	33 (61)	P=0.22
local relapse	0	10 (18)	
distant relapse	2 (14)	9 (16)	
both local and distant	2 (14)	4 (7)	
unknown location	2 (14)	10 (18)	
Death without relapse	8 (57)	21 (39)	
**Landmark 2**			
**Number (N)**	**16**	**36**	
Any CNS Relapse	7 (44)	23 (62)	P=0.17
local relapse	0	10 (27)	
distant relapse	2 (13)	5 (14)	
both local and distant	1 (6)	3 (8)	
unknown location	4 (25)	5 (14)	
Death without relapse	9 (56)	13 (36)	

[1] Chi-square test compares proportion with death without documented relapse vs any CNS progression between Early Progression and Non-Early Progression groups

Supplementary Table 3: Cause of Death Among Groups Defined by Early vs. Non-early Systemic Disease Progression at Each Landmark.

Study Groups	Cause of Death					Fisher's Exact P-value
	Systemic	CNS only	Systemic + CNS	Other	Total	
**Landmark 1**						
Non-Early Progression	15 (34)	14 (32)	8 (18)	7 (16)	44	0.86
Early Progression	7 (47)	4 (27)	2 (13)	2 (13)	15	
Total	22 (37)	18 (31)	10 (17)	9 (15)	59	
**Landmark 2**						
Non-Early Progression	8 (27)	11 (37)	5 (17)	6 (20)	30	0.44
Early Progression	7 (37)	6 (32)	5 (26)	1 (5)	19	
Total	15 (31)	17 (35)	10 (20)	7 (14)	49	

Supplementary Table 4: Among Patients Without CNS Failure (As Defined by the Study) Distribution of Patients With/Without Radiological Evidence of CNS Progression.

Study Groups	Radiological CNS Progression			Fisher's Exact P-value
	Yes	No	Total	
**Landmark 1**				
Non-Early Progression	9 (26)	25 (74)	34	>0.99
Early Progression	2 (25)	6 (75)	8	
Total	11 (26)	31 (74)	42	
**Landmark 2**				
Non-Early Progression	5 (19)	21 (81)	26	0.08
Early Progression	5 (56)	4 (44)	9	
Total	10 (29)	25 (71)	35	

* Fisher's Exact Test

Supplementary Figure 1: Depiction of Study Groups in Context of Landmarks, Stereotactic Radio-surgery and CNS Failure Free Survival.

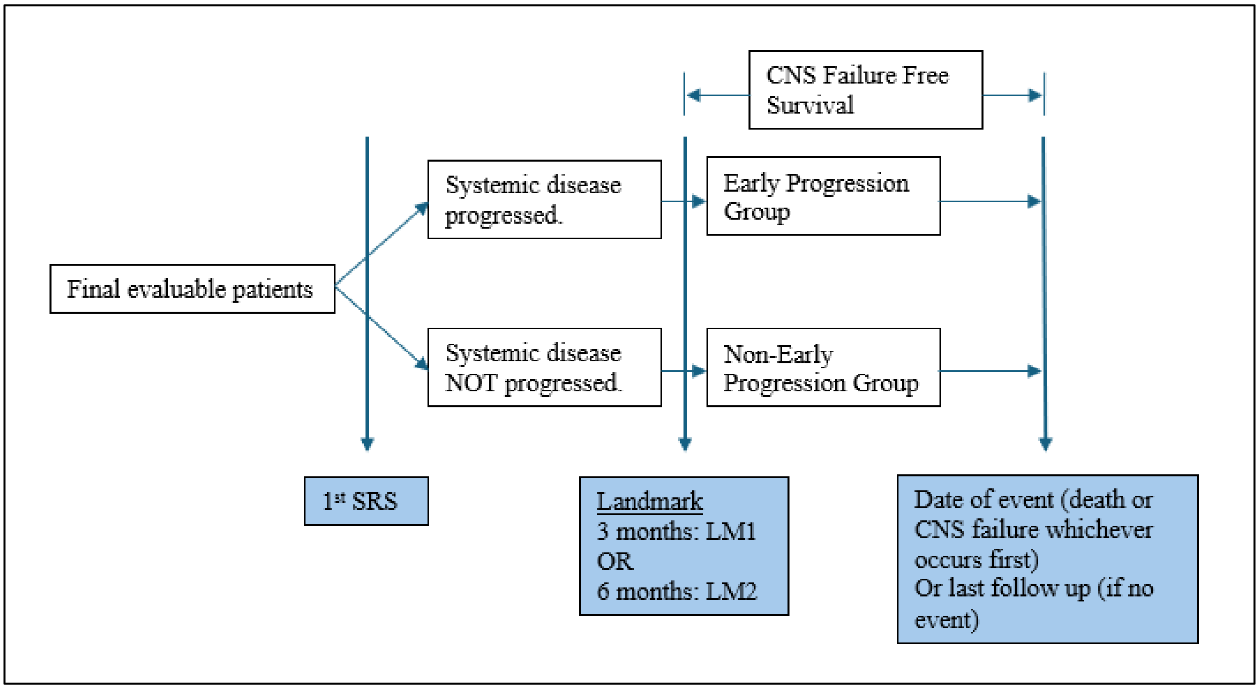

Supplementary Figure 2: CNS failure-free survival among early progression group vs. non-early progression group subdivided by the type of breast cancer (landmark 1 analysis).

Early progression group is shown as the blue line, non-early progression group red line.

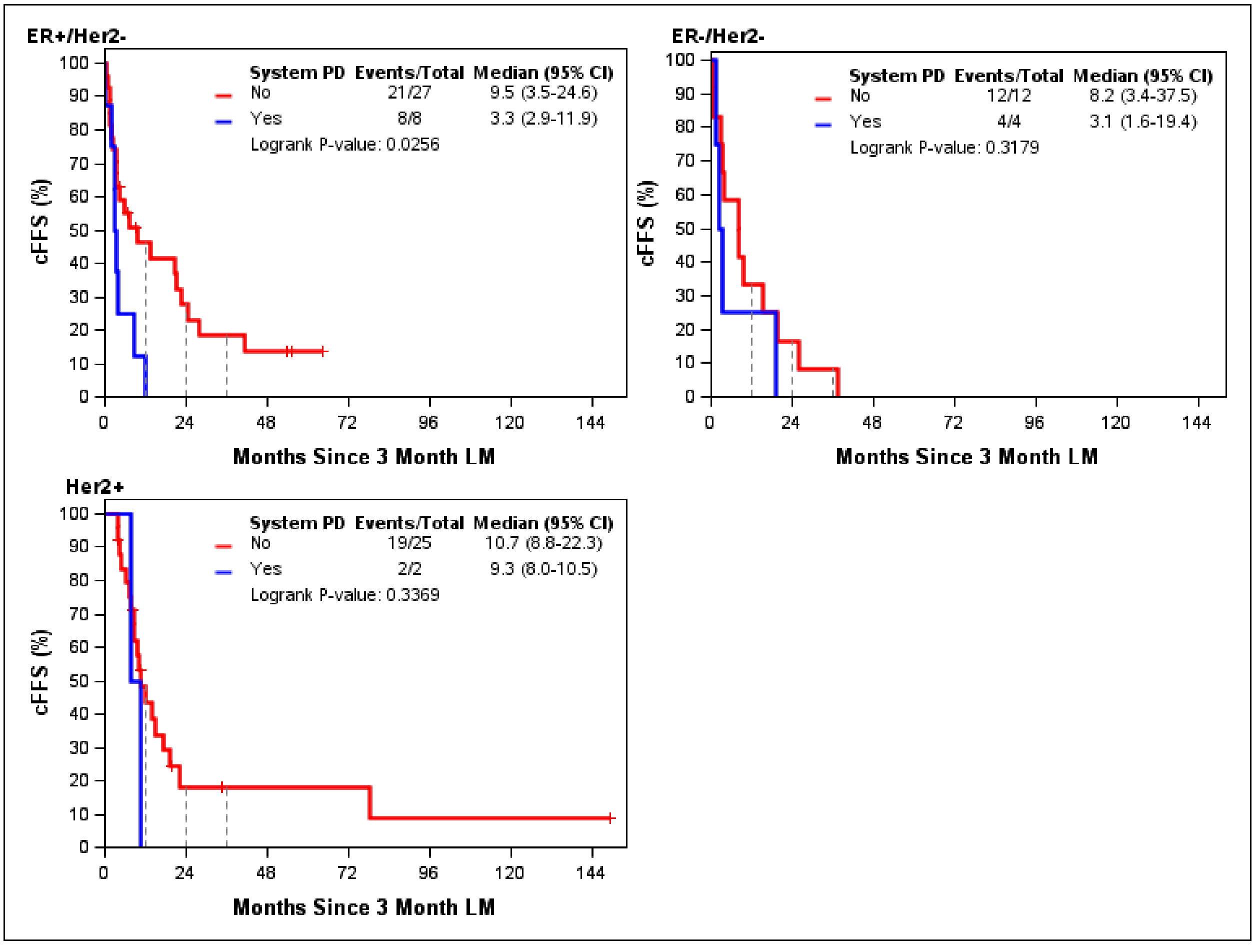

Supplementary Figure 3: Overall survival among early progression group vs. non-early progression group subdivided by the type of breast cancer (landmark 1 analysis).

Early progression group is shown as the blue line, non-early progression group red line.

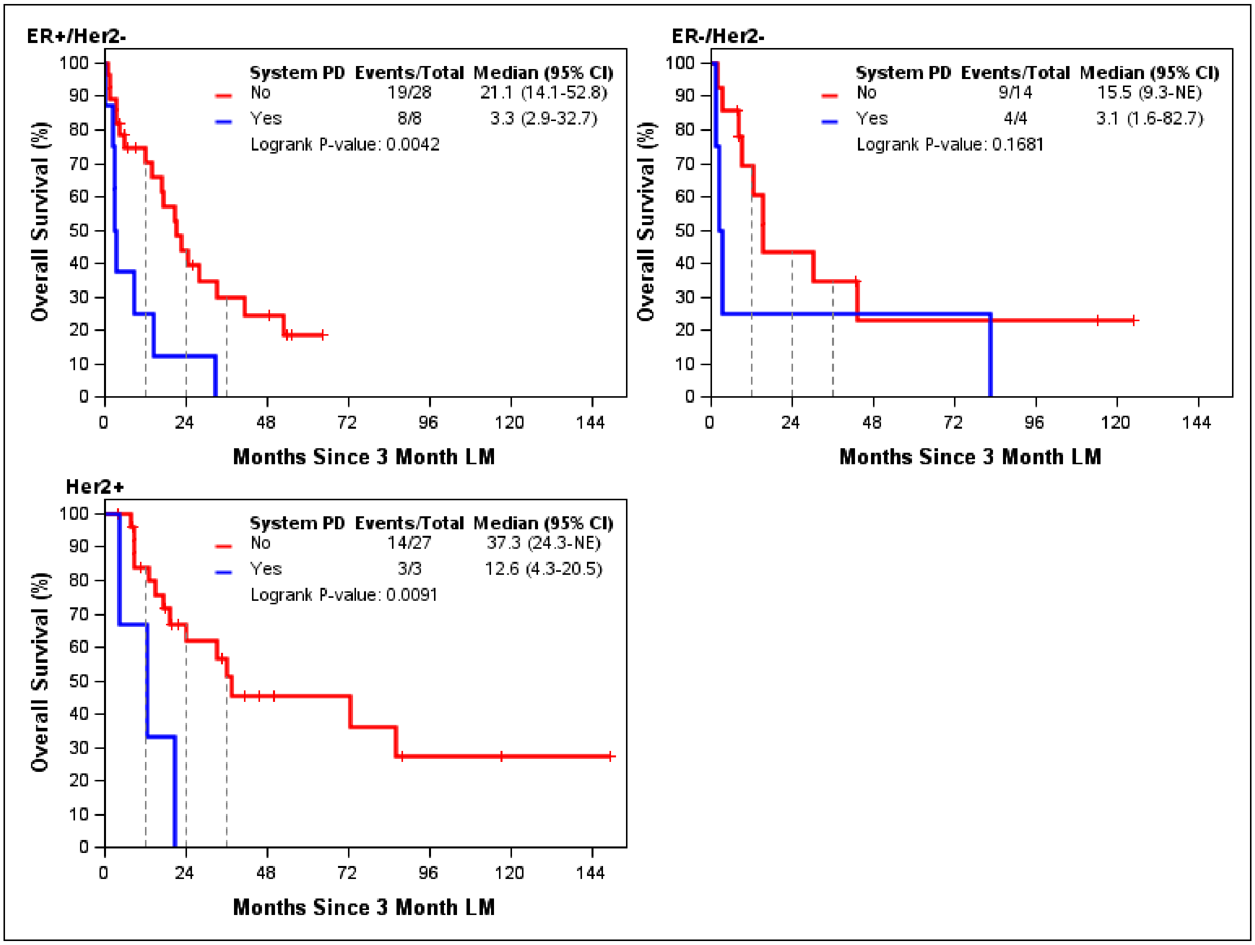

Supplementary Figure 4: CNS failure-free survival among early progression group vs. non-early progression group subdivided by the type of breast cancer (landmark 2 analysis).

Early progression group is shown as the blue line, non-early progression group red line.

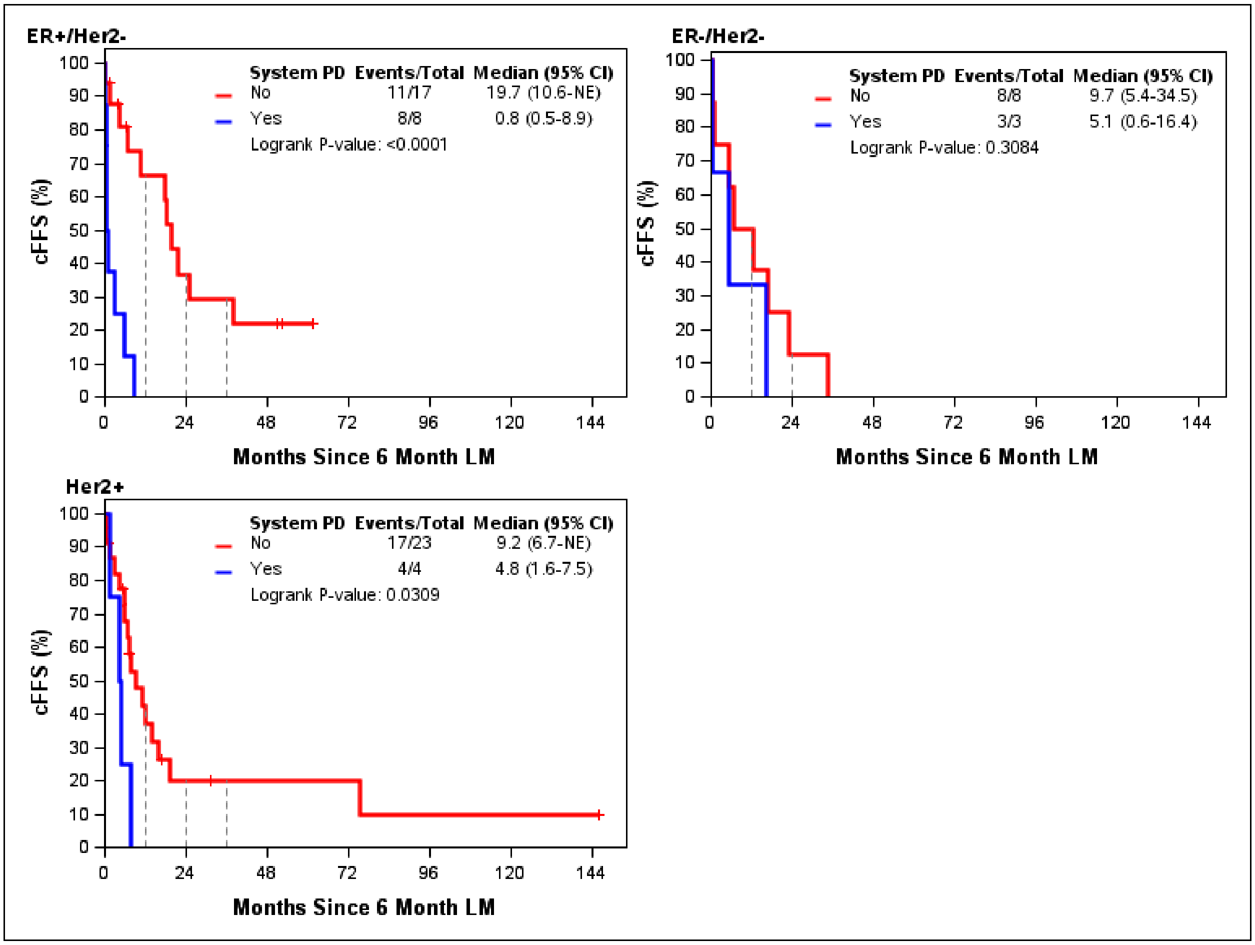

Supplementary Figure 5: Overall survival among early progression group vs. non-early progression group subdivided by the type of breast cancer (landmark 2 analysis).

Early progression group is shown as the blue line, non-early progression group red line.

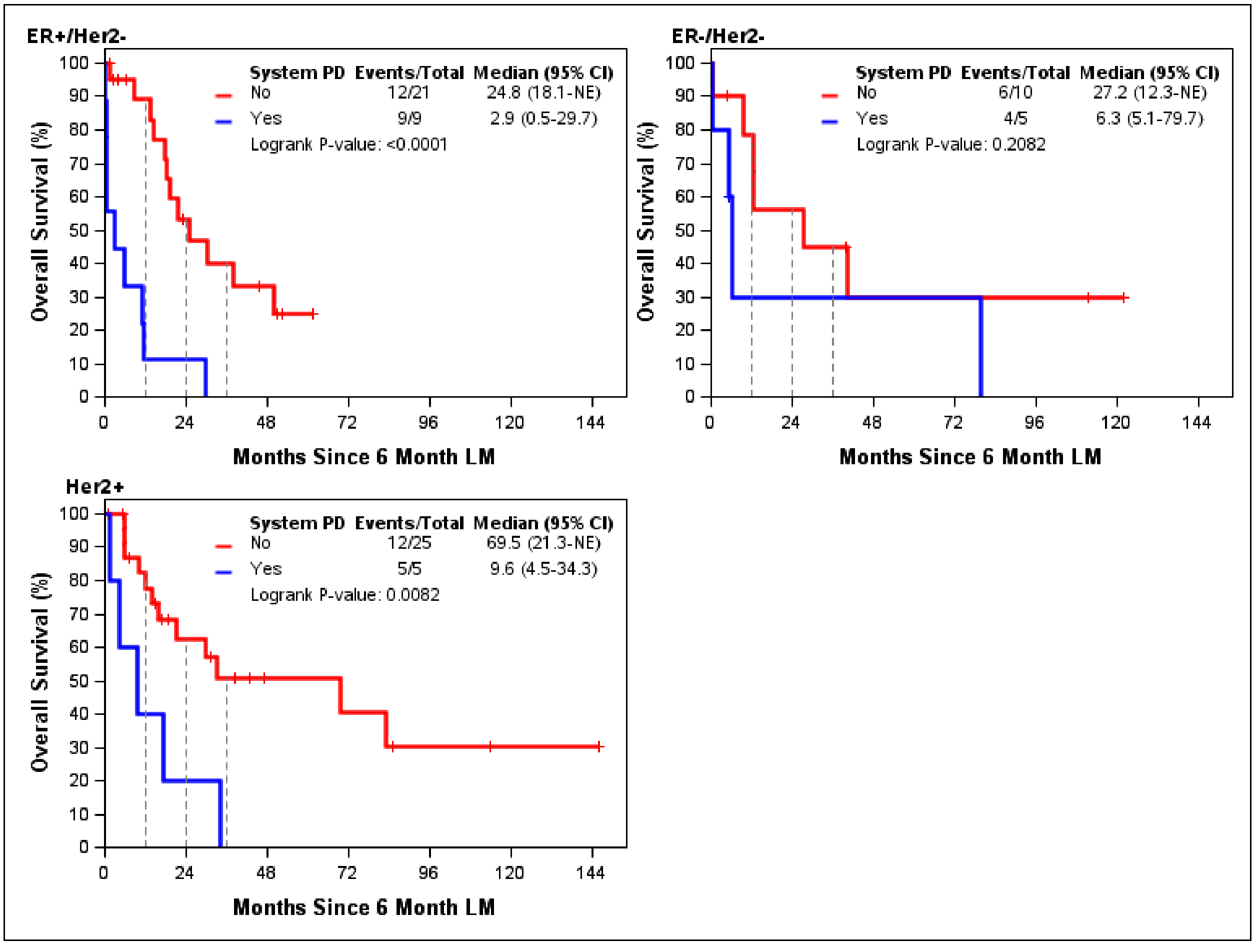

Supplementary Figure 6: CNS Failure (Including Untreated Radiological Progressions) Free Survival from landmark 1 (Figure A) and landmark 2 (Figure B):

Early progression group is shown as the blue line, non-early progression group red line.

Figure A:

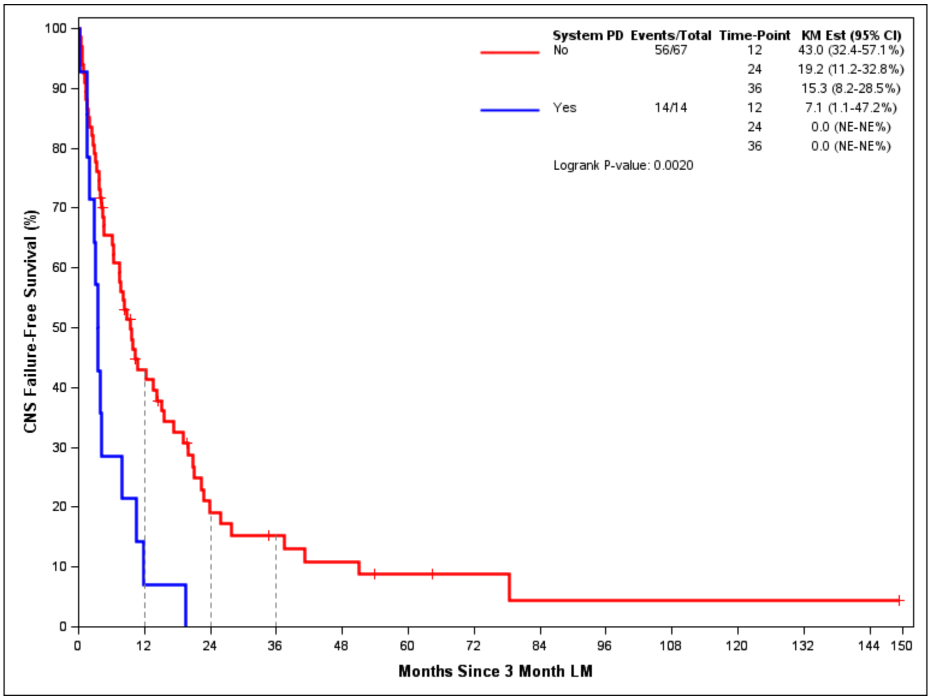

Figure B:

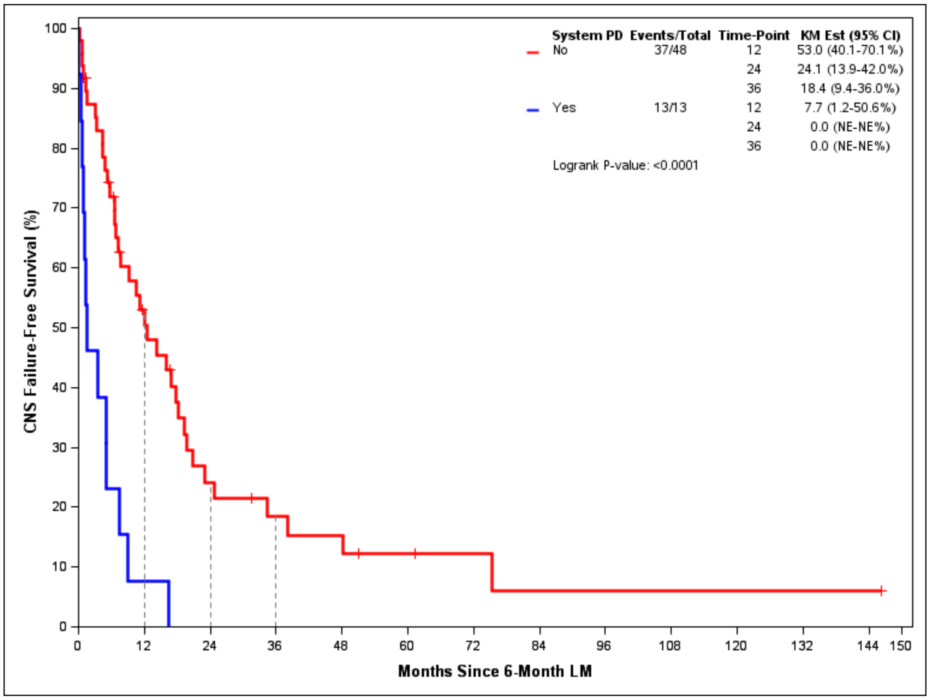

Supplementary Figure 7: Cause Specific Time to CNS Progression from landmark 1 (Figure A) and landmark 2 (Figure B):

Early progression group is shown as the blue line, non-early progression group red line.

Figure A:

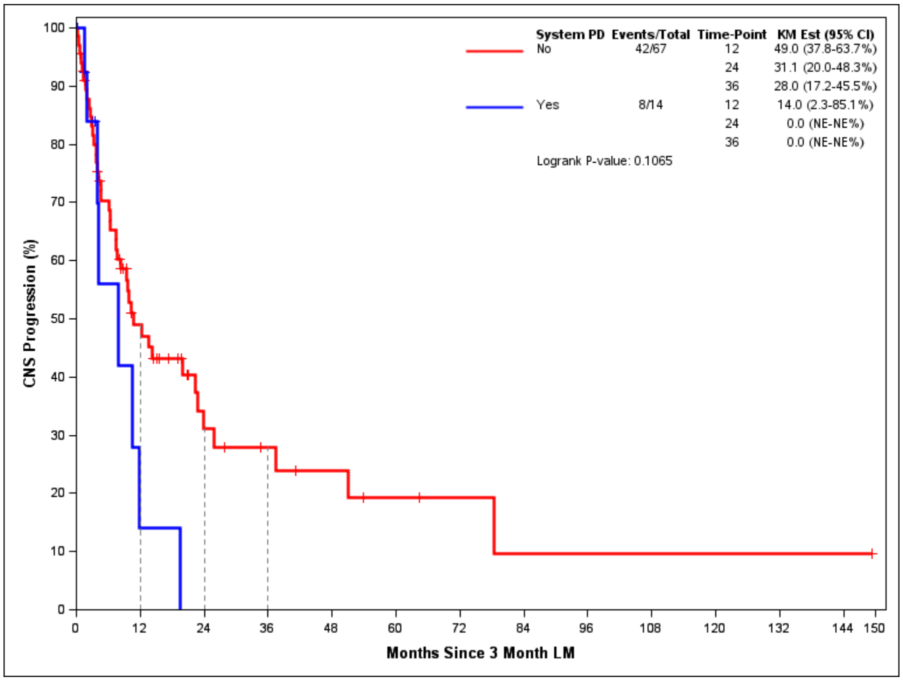

Figure B:

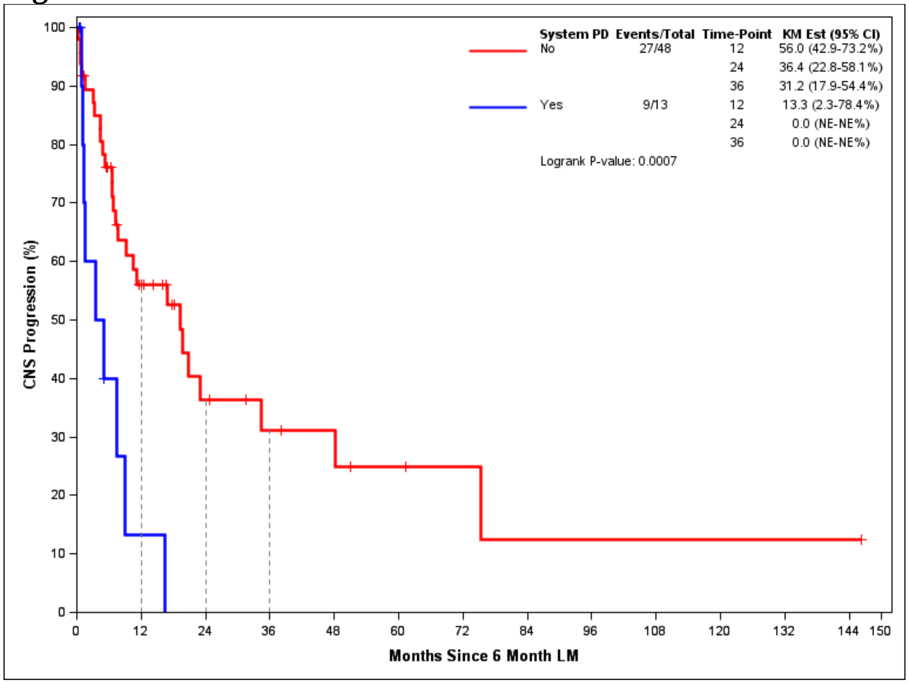

Supplement: Supplementary file 1 — Supplementary material [file 41523_2024_673_MOESM1_ESM.pdf]
